# Differential Degradation of TRA2A and PYCR2 Mediated by Ubiquitin E3 Ligase E4B

**DOI:** 10.3389/fcell.2022.833396

**Published:** 2022-05-20

**Authors:** Yao Lu, Bo Jiang, Kangli Peng, Shasha Li, Xiangnan Liu, Bufan Wang, Yuntian Chen, Tiepeng Wang, Bo Zhao

**Affiliations:** ^1^ Engineering Research Center of Cell and Therapeutic Antibody, Ministry of Education, and School of Pharmacy, Shanghai Jiao Tong University, Shanghai, China; ^2^ Department of Hand and Foot Surgery, The Second Affiliated Hospital of Soochow University, Suzhou, China; ^3^ State Key Laboratory of Radiation Medicine and Protection, Soochow University, Suzhou, China; ^4^ Department of Respiratory and Critical Care Medicine, Shanghai Ninth People’s Hospital, Shanghai Jiao Tong University School of Medicine, Shanghai, China; ^5^ National Laboratory of Biomacromolecules, Chinese Academy of Sciences Center for Excellence in Biomacromolecules, Institute of Biophysics, Beijing, China

**Keywords:** ubiquitin, ubiquitination, degradation, E4B, TRA2A, PYCR2

## Abstract

E4B belongs to the U-box E3 ligase family and functions as either an E3 or an E4 enzyme in protein ubiquitination. Transformer2A (TRA2A) and Pyrroline-5-carboxylate reductase 2 (PYCR2) are related to cancer development and are overexpressed in many cancer cells. The degradation of TRA2A and PYCR2 mediated by the ubiquitin-proteasome system (UPS) has not been reported. This study validated that E4B could ubiquitinate TRA2A and PYCR2 as an E3 ligase both *in vitro* and in the HEK293 cells. E4B mediated the degradation by forming K11- and K48- linked polyubiquitin chains on TRA2A and PYCR2, respectively. E4B regulated the alternative splicing function of TRA2A and affected RSRC2 transcription in the HEK293 cells. Although E4B is highly expressed, it hardly degrades TRA2A and PYCR2 in hepatocellular carcinoma (HCC) cells, suggesting other mechanisms exist for degradation of TRA2A and PYCR2 in the HCC cells. We finally reported that E4B interacted with substrates *via* its variable region.

## Introduction

The ubiquitin-proteasome system (UPS) is responsible for the selective degradation of short-lived proteins and plays an essential role in biological function regulation in eukaryotic cells (Hershko and Ciechanover, 1998). Substrates are targeted by ubiquitin (Ub) through an E1–E2–E3 enzymatic cascade forming different ubiquitin chains at one or more lysine residues (Komander and Rape, 2012). E4B (also called UBE4B) belongs to the U-box E3 ligase family and contains a unique U-box catalytic domain composed of 70 amino acids (Hatakeyama et al., 2001). Initially, E4B is known for its E4 function in targeting the substrates and elongating the polyubiquitin chains (Koegl et al., 1999). Subsequent studies prove that E4B manifests E3 ligase activity depending on the U-box domain, which is responsible for E2 recognition (Hoppe, 2005). E4B is essential in embryo survival and cardiac and nervous system development during the stages of embryonic development (Kaneko-Oshikawa et al., 2005; Mammen et al., 2011; Wu and Leng, 2011). Intriguingly, the alteration of the gene or protein of E4B is involved in the genesis of neuropathies and various types of cancer. E4B has been found to be either overexpressed or suppressed in different cancer such as breast cancer, hepatocellular carcinoma, glioblastoma, promyelocytic leukemia, colorectal cancer, and neuroblastoma (Krona et al., 2003; Heuze et al., 2008; Wu et al., 2011; Zhang et al., 2014; Zhang et al., 2016). Additionally, E4B can regulate the p53 level to inhibit cell apoptosis and promote tumor development through two pathways. As an E4, E4B synergistically collaborates with MDM2 to target p53 for its ubiquitination and degradation (Fang et al., 2000; Wu et al., 2011). E4B can negatively regulate the protein levels of phosphorylated p53 at Ser15 and Ser392, independent of MDM2 (Du et al., 2016). So far, the mechanism of how E4B affects tumorigenesis is unclear. Except for p53, there is lacking indepth knowledge on other substrates ubiquitination mediated by E4B. Ufd2p, the homolog of E4B in *S. cerevisiae*, contains an N-terminal variable region, a highly conserved Ub elongating region, and a C-terminal U-box domain (Tu et al., 2007). However, the full-length structure of E4B is still not well characterized due to its complexity in the N-terminal variable region (Nordquist et al., 2010). The interaction of E4B with its ubiquitination substrates is also reported rarely.

In our previous work, we developed an orthogonal ubiquitin transfer (OUT) method to identify the substrates of E4B (Zhao et al., 2012; Bhuripanyo et al., 2018; Zhao et al., 2020). In this method, over 100 proteins were attached by xUb (an Ub mutant) under the transfer of E4B in the HEK293 cells, indicating that these proteins may be the potential ubiquitination substrates of E4B (Bhuripanyo et al., 2018). Among these proteins, Transformer2A (TRA2A) and Pyrroline-5-carboxylate reductase 2 (PYCR2) are closely related to cancer development. TRA2A and TRA2B, different isoforms of TRA2 in humans, are encoded by the Transformer2 gene and participate in the alternative splicing (AS) process of pre-mRNA to produce multiple mature mRNAs (Best et al., 2014). Both TRA2A and TRA2B mediate splicing events to affect tumor progression and drug sensitivity (Hirschfeld et al., 2011; Best et al., 2014; Tieju Liu et al., 2017). TRA2A is overexpressed in the glioma cells and triple-negative breast cancer to promote proliferation, invasion, migration, and epithelial–mesenchymal transition (Tieju Liu et al., 2017; Tan et al., 2018). TRA2A can reduce the level of normal RSRC2 splicing product RSRC2s and increase the expression of RSRC2l, an abnormal mRNA splicing product of RSRC2, which promotes the progression of triple negative breast cancer (Tieju Liu et al., 2017). PYCR2, an enzyme that catalyzes the synthesis of l-proline from Δ1-pyrroline-5-carboxylate (P5C), was reported to promote cancer growth and inhibit apoptosis through multiple approaches, such as regulation of cell cycle and redox homeostasis and promotion of growth signaling pathways (Meng et al., 2017; Escande-Beillard et al., 2020; Li et al., 2021). As metabolic reprogramming has been considered a new sign of cancer in recent years, proline metabolism is believed to be a critical factor in the cancer cell growth (Pavlova and Thompson, 2016). The change of proline expression is the most significant factor in amino acid metabolism in hepatocellular carcinoma (HCC), and PYCR2 is abnormally expressed in the esophageal squamous cell carcinoma (ESCC), indicating that PYCR2 may play a key role in cancer progression (Tang et al., 2018; Sun et al., 2019).

The ubiquitination of TRA2A and PYCR2 has not been reported. In this study, we verified that E4B could ubiquitinate TRA2A and PYCR2 as an E3 ligase both *in vitro* and in the HEK293 cells. E4B mediated the formation of K11- or K48- linked polyubiquitin chains on TRA2A and PYCR2 and induced their degradation by the proteasome. Regulation of E4B affected the alternative splicing function of TRA2A in the HEK293 cells. However, the degradation of TRA2A and PYCR2 in the HCC cells was quite different from that in the HEK293 cells, indicating there are other mechanisms for the degradation of TRA2A and PYCR2 in the HCC cells. By constructing the variants of E4B, we validated that the variable region of E4B is an indispensable domain for the interaction of E4B with its substrates, TRA2A and PYCR2.

## Materials and Methods

### Cell Culture and Reagents

DMEM (10100147) and fetal bovine serum (C11995500BT) were from Gibco. MG132 (HY-13259) and protease-inhibitor cocktail (HY-K0010) were from MCE. Lipofectamine™ 3000 Transfection Reagent (L3000015) was from Invitrogen. Cycloheximide (CHX) and N-ethylmaleimide (NEM) were from Sigma-Aldrich. The following antibodies were from Abcam: anti-E4B (ab126759), anti-ubiquitin (ab134953), anti-HA (ab182009). Anti-GAPDH antibody (60004-1-Ig) and anti-PYCR2 antibody (17146-1-AP) were from Proteintech. Anti-TRA2A antibody (GTX87998) was from GeneTex. Anti-MYC antibody (9B11) was from Cell Signaling Technology. Anti-FLAG (M2) antibody (F1804) and anti-FLAG(M2) affinity gel (A2220) were from Sigma-Aldrich. Goat anti-rabbit IgG-Alexa Fluor 790 antibody (111-655-144) and goat anti-mouse IgG- Alexa Fluor 790 antibody (115-005-072) were from Jackson ImmunoResearch. Protein G Agarose (16-266) was from Merckmilipore. Phenylmethanesulfonyl fluoride (PMSF, ST506) and RIPA Lysis Buffer (P0013C) were from Beyotime. Polyetherimide (AC04L091) was from Life-iLab.

### Plasmids and Small Interfering RNA Oligonucleotides

Full-length human TRA2A and PYCR2 were cloned into FLAG-tagged pcDNA 3.1 vector. The full-length Ub was cloned into HA-tagged pcDNA 3.1 vector. The genes of Ub mutants, K11R, K48R, and K63R, were synthesized by GENEWIZ. Myc-tagged full-length human E4B and variants were cloned into pLVX-IRES-mcherry vector. His-tagged TRA2A, PYCR2, Ub, and E4B were cloned into pET-28a + vector for protein expression. All plasmids were verified by sequencing. GIPZ Lentiviral shRNA pGIPZ-shE4B and pGIPZ-empty were from Dharmacon**.**

Small interfering RNA (siRNA) targeted TRA2A was generated by Sangon Biotech. The sequence of **siTRA2A** was forward: 5′-GCC​UCA​GUU​UGU​ACA​CAA​CTT-3′ and reverse: 5′-GUU​GUG​UAC​AAA​CUG​AGG​CT-3’.

### Recombinant Protein Purification and *In Vitro* Ubiquitination Assay

His and FLAG-tag TRA2A and PYCR2 were transformed into BL21 *E. Coli* cells for protein expression. The cells were grown for 4–8 h at 37°C until the OD was 0.8. IPTG (0.25 μM) was added to the cells, and the expression was induced at 16°C overnight. The cell cultures were spun down and lysed in lysis buffer (50 mM Tris-Base, 500 mM NaCl, 5 mM imidazole, and pH 8.0). Ni-NTA was incubated with the cell lysates for 2 h to bind the target proteins. The cell lysates were washed several times and the elution was collected to obtain the purified targeted proteins.

To assay ubiquitination of these substrates by E4B, 10 μM FLAG-tagged substrate proteins were incubated with 14 μM Ub, 1 μM E1 (Ube1), 5 μM E2 (UbcH5b), and 1 μM E3 (E4B) in TBS buffer containing 50 mM MgCl_2_ and 1.5 mM ATP. After a 2-h ubiquitin transferring reaction at room temperature, ubiquitination was detected by immunoblotting with an anti-FLAG antibody.

### Cells Culture and Transfection

HEK293, HepG2, and HuH7 were cultured in DMEM supplemented with 10% fetal bovine serum and incubated in a 37°C humidified incubator with 5% CO_2_. ShE4B and shctrl cells were cultured in the same culture medium with an extra 0.2 μg/ml puromycin. HEK293 cells were cotransfected with the indicated plasmids using the linear Polyetherimide (PEI) reagent according to the manufacturer’s protocol. HEK293 was transfected with a small interfering RNA (siRNA) to knock down TRA2A with Lipo3000 according to the manufacturer’s protocol. HepG2 and HuH7 cells were transfected with Lipo3000 according to the manufacturer’s protocol.

### *In Vivo* Ubiquitination and Co-Immunoprecipitation

The cells were incubated with 10 μM MG132 for 4 or 6 h before harvest. For normal ubiquitination assay, the cells were lysed with RIPA lysis buffer 1 (50 mM Tris (pH 7.4), 150 mM NaCl, 1% NP-40, 0.5% deoxycholate, 0.1% SDS) containing protease inhibitor cocktail (PIC) and Phenylmethanesulfonyl fluoride (PSMF) for 48 h after transfection. For the ubiquitin chains assays, an extra 100 μM N-ethylmaleimide (NEM) and 100 μM O-Phenanthroline were added to the RIPA lysis buffer 1. For the interaction of E4B and substrates assays, the cells were lysed with RIPA lysis buffer 2 (50 mM Tris (pH 7.4), 150 mM NaCl, 1% NP-40) containing protease inhibitor cocktail (PIC) and Phenylmethanesulfonyl fluoride (PSMF) for 48 h after transfection. For the immunoprecipitation, 15 μl anti-FLAG (M2) affinity gel for each sample was washed with 0.5 ml of cold TBS three times, then it was added to 1 mg of cell lysates. Shake all the samples gently at 4°C for 4 h, then centrifuge the resin for 1 min at 7,000×*g*. Wash the beads three times with 0.5 ml of cold TBS-T. After the final wash, remove the wash buffer and add 30 μl TBS and 9 μl 5×SDS loading. Centrifuge the samples at 10,000×*g* for 1 min and boil the samples for 15 min. The ubiquitination of the substrates was assayed by immunoblotting with an anti-Ub antibody under denaturing conditions. The ubiquitination chains of substrates were assayed by immunoblotting with an anti-HA antibody under denaturing conditions. The protein–protein interaction between the substrates and E4B was assayed by immunoblotting with an anti-MYC antibody under nondenaturing conditions.

### Degradation of Substrates in HEK293 and HCC Cells

1 × 10^6^ HEK293 cells were seeded into 6-well plates and were transfected with FLAG-tagged substrate plasmids and increasing amounts of pLVX-E4B (0, 0.5, 1, 2, and 3 μg) plasmids by PEI. The cells were lysed with RIPA lysis buffer after transfection for 48 h. The amount of total substrate proteins was determined by immunoblotting with an anti-FLAG antibody. HepG2, HuH7, and HEK293 cells were transfected with increasing pLVX-E4B (0, 1, and 2 μg) plasmids with Lipo3000.

For CHX chase assays, 1 × 10^6^ HEK293 cells were seeded into 6-well plates and were cotransfected with 1.5 μg substrate plasmids and 1 μg pLVX-E4B or 1 μg empty pLVX. Another group was only transfected with 1.5 μg substrate plasmids to shE4B and shctrl, respectively. The cells were harvested 48 h after transfection. To block *de novo* protein synthesis, the cells were treated with Cycloheximide (CHX) (50 μg/ml) and incubated for 0, 2, 4, 6, and 8 h before harvesting the cells. The amount of total substrate proteins was determined by immunoblotting with an anti-FLAG antibody. CHX chase assays were performed in the HEK293 cells that stably expressed anti-E4B shRNA to determine the effect of shE4B on substrate stability.

### RNA Extraction and RSRC2 Splicing Assays

2.5 × 10^5^ HEK293 cells were seeded into 12-well plates and were transfected with different amounts of FLAG-tagged TRA2A plasmids or E4B plasmids. The total RNA from 12 well-plates was extracted by 500 μl TRIzol reagent after 48 h transfection. RNA was isolated for subsequent RT-PCR to obtain a cDNA library. GAPDH gene was amplified as a control. The primers used in this study were shown below:

RSRC2 forward: 5′-AGA​AAA​CAC​AGG​AGC​CGG​AG-3′.

RSRC2 reverse: 5′-TGA​GTG​ACT​TCT​GCC​TCT​TGA-3′.

GAPDH forward: 5′-TCA​AGA​AGG​TGG​TGA​AGC​A-3′.

GAPDH reverse: 5′-AAG​GTG​GAG​GAG​TGG​GT-3′.

### Statistical Analysis

A statistical analysis was performed using Graphpad Prism (Graphpad prism 8.0 software, San Diego, CA, United States). All the quantitative data were presented as the mean ± SD. Other statistical analysis was performed using the unpaired Student’s t-test. *p* < 0.05 was considered statistically significant.

## Results

### E4B Mediated TRA2A and PYCR2 Ubiquitination Both *in Vitro* and *in Vivo*

**FIGURE 1:**
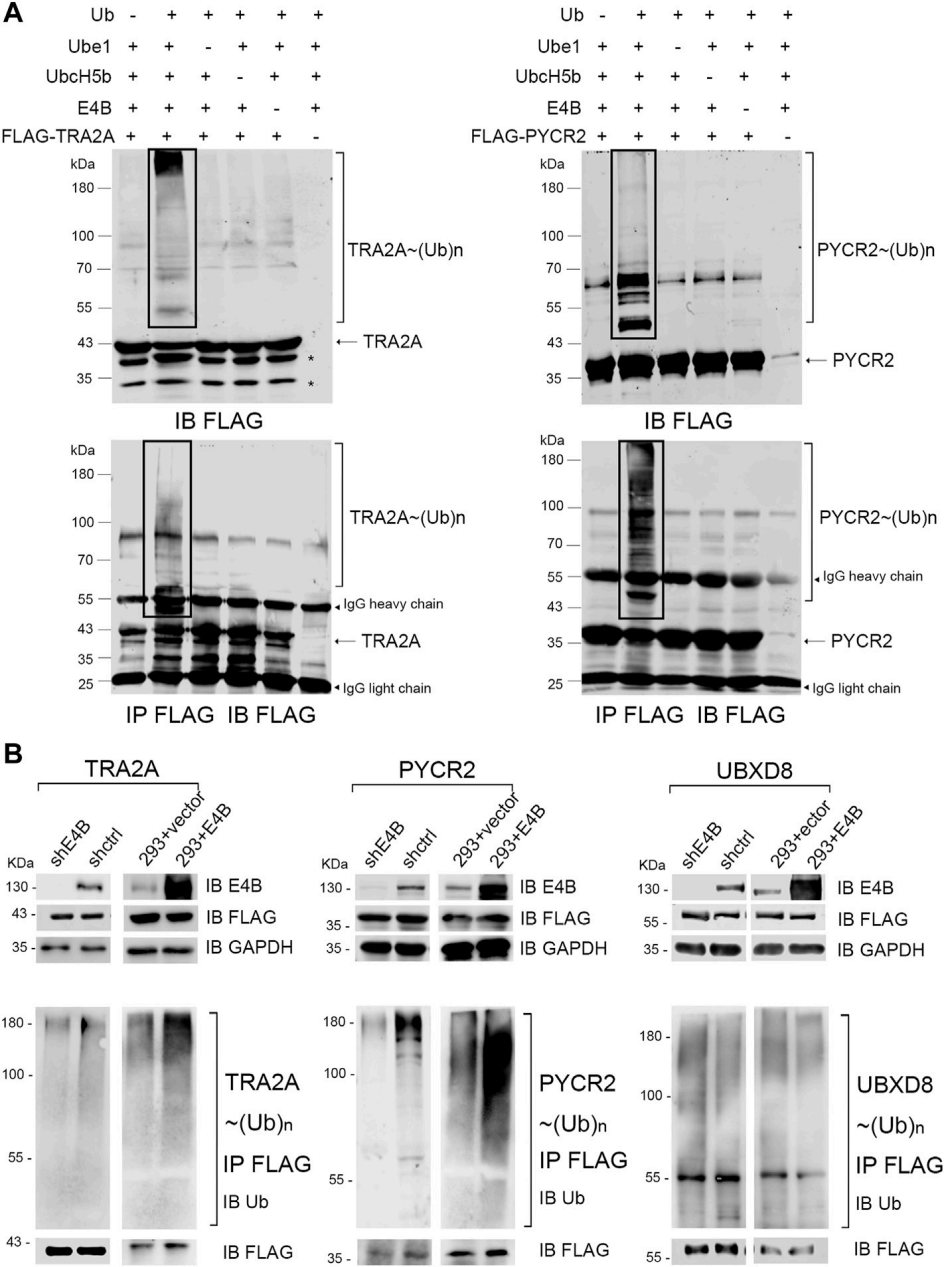
E4B mediated TRA2A and PYCR2 ubiquitination *in vitro* and *in vivo*. **(A)** *In vitro* ubiquitination of TRA2A and PYCR2 through Ube1-UbcH5b-E4B cascade (line 2) and controls without Ub (line 1), E1 (line3), E2 (line 4), E3 (line 5), and FLAG-tagged substate (line 6) were detected with an anti-FLAG antibody. **(B)** Ubiquitination of TRA2A and PYCR2 were detected in the HEK293 cells. One group was stably E4B knockdown HEK293 cell (shE4B) and sh-empty HEK293 cell (shctrl). Another group was the E4B overexpressed HEK293 cell (293 + E4B) and pLVX-empty HEK293 cell (293 + vector). All these groups were transfected with FLAG-tagged TRA2A or PYCR2, respectively, and incubated with MG132 (10 μM) for 4 h before harvesting. The cell lysates were pulled down by immunoprecipitation with an anti-FLAG antibody under a denaturing condition. Ubiquitination of TRA2A and PYCR2 was detected by immunoblotting with an anti-Ub antibody.

In our previous work, we employed the orthogonal ubiquitin transfer (OUT) to identify the proteins that could be targeted by xUb through an xUb-xE1-xE2-xE4B cascade in the HEK293 cells (Zhao et al., 2012; Bhuripanyo et al., 2018). We believe that these are the potential ubiquitination substrates of E4B. Two important proteins: TRA2A and PYCR2, appeared in the OUT pathway based on the MS analysis. Here, we want to verify if TRA2A and PYCR2 are bonafide substrates of E4B. We first expressed and purified FLAG-tagged TRA2A and PYCR2 proteins in *E. coli* cells and established an *in vitro* ubiquitination reaction by mixing with HA-Ub, E1 (Ube1), E2 (UbcH5b), and E3 (E4B) recombinant proteins. Ubiquitination of TRA2A and PYCR2 was detected by immunoblotting with an anti-FLAG antibody. We observed significant polyubiquitination formed on TRA2A and PYCR2 in the presence of all the elements for ubiquitin transfer (Figure 1A, line 2). In contrast, neither mono- nor poly-ubiquitination were observed if there was a lack of any enzyme or Ub, or substrates for Ub transfer (Figure 1A). To ensure that the ubiquitination was attached to TRA2A and PYCR2, we further used anti-FLAG beads to pull down the substrates in all the groups under a denaturing condition. The immunoprecipitation results were the same as the ubiquitination in the protein reactions. To show the individual components of the *in vitro* reaction, another transfer assay was designed to detect the HA-Ub attached to components individually with an anti-HA antibody under a nondenaturing condition (Supplementary Figure S1). In this assay, HA-Ub could be attached to E1 and E2 to form a thioester bond, while E4B and the substrates can form polyubiquitin chains during the Ub transfer. These results suggested that Ub was attached to TRA2A and PYCR2 depending on the E1-E2-E3 enzymatic transfer and E4B worked as an E3 ligase for the *in vitro* ubiquitination of TRA2A and PYCR2.

We next examined whether TRA2A and PYCR2 could be ubiquitinated by E4B in the HEK293 cells. The HEK293 cells were transfected with a short hairpin RNA against E4B expression to generate a stable E4B knockdown cell line (shRNA) in our previous study. A scramble shRNA was also used to generate the shctrl cells. Meanwhile, the HEK293 cells were transfected with a pLVX-empty vector or pLVX-E4B plasmid to generate the HEK293 + vector (293 + vector) and E4B overexpressed cell line (293 + E4B). E4B knockdown and overexpression groups were both transfected with pcDNA-FLAG-TRA2A and pcDNA-FLAG-PYCR2 plasmids, respectively. Meanwhile, pcDNA-FLAG-UBXD8 was used as a negative control because UBXD8 could not be ubiquitinated by E4B in our previous study (data not shown). The cells were treated with proteasome inhibiter MG132 for 4 h before harvesting. The expression level of E4B was detected with an anti-E4B antibody. FLAG-tagged TRA2A, PYCR2, and UBDX8 were detected with an anti-FLAG antibody. TRA2A, PYCR2, and UBDX8 were pulled down with the anti-FLAG beads and immunoblotted with an anti-Ub antibody to detect the ubiquitination. Significant polyubiquitinations on TRA2A and PYCR2 were observed in 293 + E4B cells compared to 293 + vector cells (Figure 1B). In contrast, the ubiquitination of TRA2A and PYCR2 in the shRNA cells decreased compared to that in the shctrl cells. However, there is no difference in ubiquitination of UBDX8 in all the groups, indicating that the ubiquitination of UBDX8 was not affected by E4B. These results showed that TRA2A and PYCR2 could be ubiquitinated by E4B in the HEK293 cells, and the ubiquitination depended on the level of E4B in the HEK293 cells.

### E4B Promoted the Degradation of TRA2A and PYCR2 by Forming K11- or K48- Linked Polyubiquitin Chains

**FIGURE 2:**
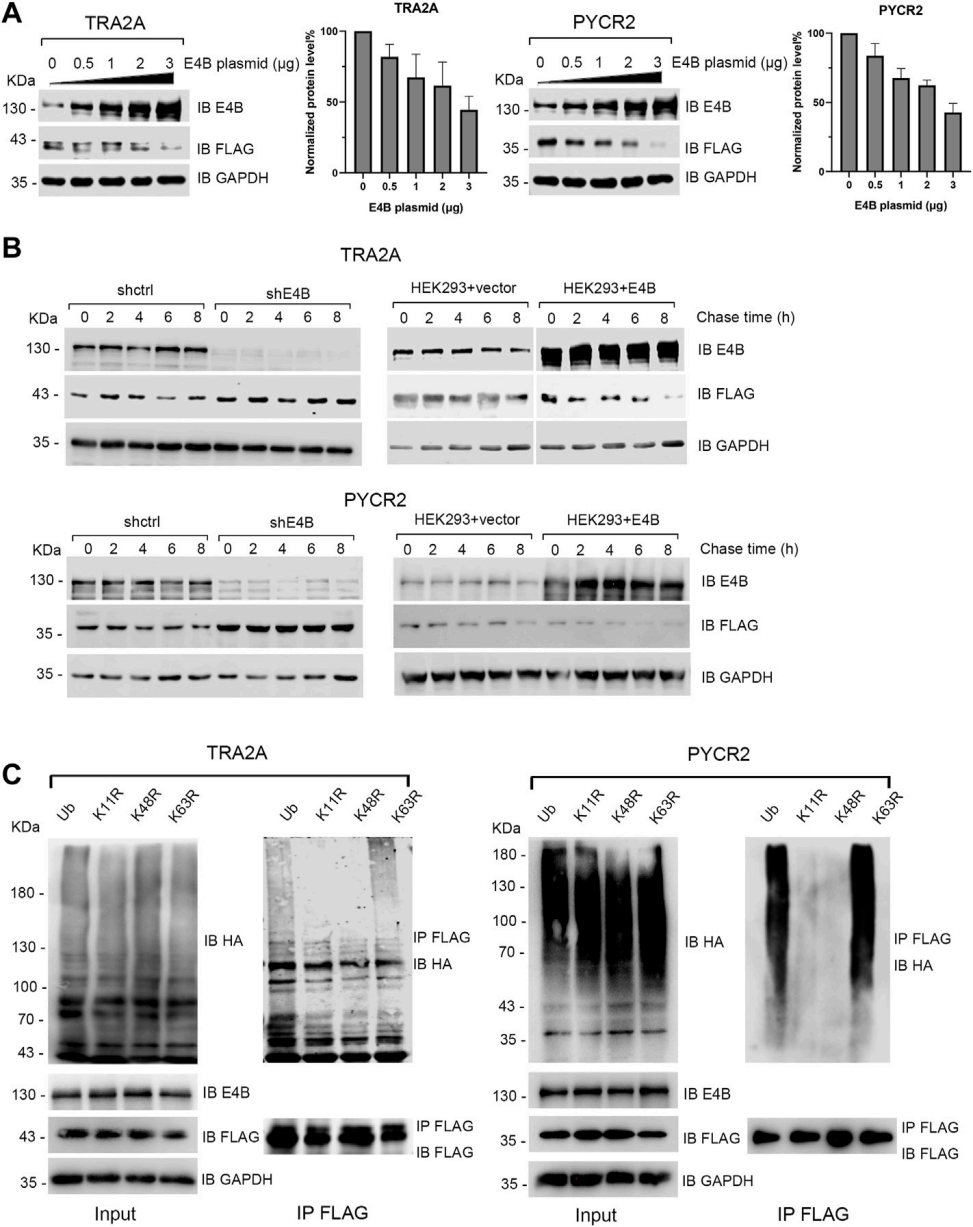
E4B promoted the degradation of TRA2A and PYCR2. **(A)** Increase of E4B accelerated the degradation of TRA2A and PYCR2. The HEK293 cells were transfected with different amounts of E4B plasmid but the same amount of FLAG-TRA2A and FLAG-PYCR2 plasmids, respectively. The protein levels of TRA2A and PYCR2 were detected with an anti-FLAG antibody. Line charts correspond to the WB results. Each sample was measured three times. **(B)** E4B-dependent degradation of TRA2A and PYCR2 assayed by CHX chase. One group was the E4B knockdown HEK293 cells (shE4B) and sh-empty HEK293 cells (shctrl). Another group was the E4B overexpressed HEK293 cells (HEK293 + E4B) and pLVX-empty HEK293 cells (HEK293 + vector). The protein levels of substrates in every group were detected at 0, 2, 4, 6, and 8 h after 50 μg/ml CHX was added to the cells before harvesting. Each sample was measured three times. **(C)** E4B formed K11- and K48-linked polyubiquitin chains on TRA2A and PYCR2. The HEK293 cells were transfected with HA-wtUb (line 1), HA-K11R (line 2), HA-K48R (line 3), and HA-K63R (line4) plasmids. The cells were treated with MG132 for 6 h to ensure the stability of substrates. Ubiquitination of the substrates was detected by immunoblotting with an anti-HA antibody and pulled down FLAG-substrates were detected with an anti-FLAG antibody to equal the amounts.

Since E4B could ubiquitinate TRA2A and PYCR2 *in vitro* and *in vivo*, we wanted to know whether E4B mediates the degradation of TRA2A and PYCR2. The HEK293 cells were transfected with different amounts of E4B, and the degradation of TRA2A and PYCR2 was detected in two independent experiments simultaneously without MG132 treatment. With the increasing amount of E4B, the protein levels of TRA2A and PYCR2 decreased significantly, indicating that E4B promoted the degradation of TRA2A and PYCR2 (Figure 2A). We further performed a cycloheximide (CHX) chase assay to detect the stability of TRA2A and PYCR2. The cells were treated with 50 μg/μL CHX to inhibit protein synthesis and were harvested at 0, 2, 4, 6, and 8 h after the addition of CHX to detect the protein levels of TRA2A and PYCR2 with an anti-FLAG antibody. In the shE4B cells (shE4B), the turnover of TRA2A and PYCR2 was stable even after 8 h treatment with CHX, while TRA2A and PYCR2 were both decreased after 4 h in the shctrl cells. (Figure 2B). In the HEK293 + vector groups (293 + vector), which were not transfected with exogenous E4B but pLVX-empty plasmid, the protein levels of TRA2A and PYCR2 decreased after 4 h treatment with CHX (Figure 2B). Noticeable turnover was observed in the E4B overexpressed cells (293 + E4B), in which the protein levels of TRA2A and PYCR2 decreased significantly compared to that in the 293 + vector group (Figure 2B). These results indicated that TRA2A and PYCR2 are bonafide substrates of E4B. Increasing the expression of E4B in the HEK293 cells accelerated the degradation of TRA2A and PYCR2.

Ub consists of seven Lys (K) residues that can form different types of polyubiquitin chains (Akutsu et al., 2016). Among them, K11- or K48- linked polyubiquitin chains signal protein degradation (Akutsu et al., 2016; Boughton et al., 2020). We wanted to know whether E4B formed K11- or K48- linked polyubiquitin chains on TRA2A and PYCR2. In addition, K63- linked polyubiquitin chain is known as a canonical Ub signal and has been widely studied in inflammatory signaling and NF-κB pathway (Meyer and Rape, 2014). By replacing one Lys (K) to Arg (R) in Ub, we constructed three Ub mutants: K11R, K48R, and K63R. We transfected the HEK293 cells with HA-tagged wtUb and Ub mutants and detected their expression with an anti-HA antibody (Figure 2C). Meanwhile, we cotransfected E4B and FLAG-tagged TRA2A and PYCR2 to these HEK293 cells and detected the ubiquitination of TRA2A and PYCR2 with an anti-HA antibody after MG132 (10 μM) treatment for 6 h before harvesting. The results showed that the polyubiquitination formed by K11R and K48R reduced significantly compared to wtUb or K63R (Figure 2C). These results validated that E4B formed K11- or K48- linked polyubiquitin chains on TRA2A and PYCR2 and mediated their degradation.

### E4B Regulated the Alternative Splicing Function of TRA2A and Affected the Transcription of RSRC2

**FIGURE 3:**
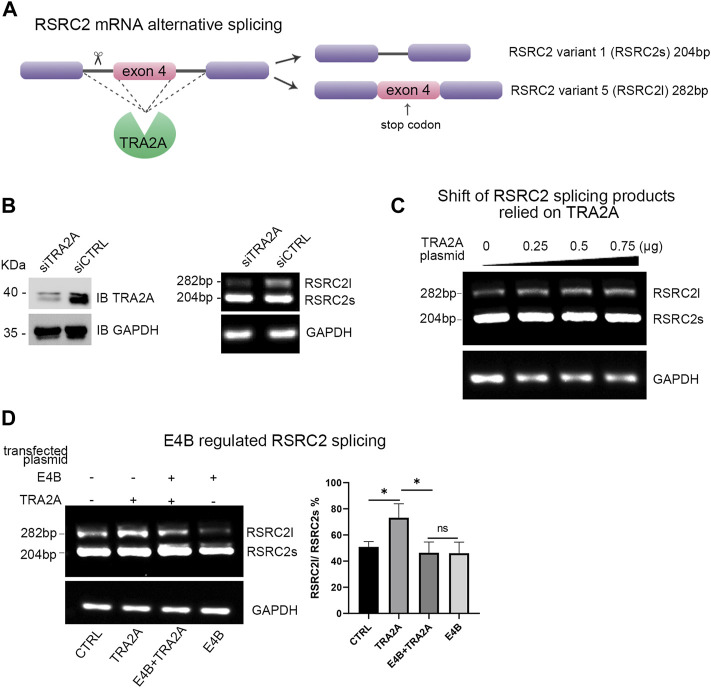
E4B affected alternative splicing of RSRC2. **(A)** Maps of RSRC2 mRNA alternative splicing regulated by TRA2A. There are two splicing variants: RSRC2s and RSRC2l. RSRC2s is the main product while RSRC2l is an abnormal splicing product with an additional exon 4 gene remaining and encoding a stop codon. **(B)** Knockdown TRA2A deceased the abnormal splicing product RSRC2l. Endogenous protein level of TRA2A was exhausted after being transfected with siTRA2A and the total RNA was extracted to generate the cDNA library. RSRC2 gene was amplified by a pair of specific primers and GAPDH gene was amplified as an intracellular control. **(C)** Overexpression of TRA2A induced the shift of RSRC2 splicing products from RSRC2s to RSRC2l. The HEK293 cells were transfected with 0, 0.25, 0.5, and 0.75 μg TRA2A plasmid, respectively, and the total RNA was extracted to generate the cDNA library. RSRC2 gene was amplified by a pair of specific primers and GAPDH gene was amplified as an intracellular control. **(D)** Alternative splicing of RSRC2 was regulated by the expression of TRA2A and E4B. 0.75 μg TRA2A and 1 μg E4B plasmids were transfected into the HEK293 cells in different groups. The bar chart was drawn according to the yields of PCR products. All the experiments were repeated three times. (*p < 0.5 vs. control; ns: *p* > 0.05)

TRA2A regulates multiple alternative splicing (AS) events by processing the mRNA precursors to mature mRNA (Best et al., 2014). Arginine and serine rich coiled-coil 2 (RSRC2) has been reported to be spliced into two variants by TRA2A at the mRNA level (Tieju Liu et al., 2017). The shorter variant (variant 1, also named RSRC2s) encodes a functional protein, while the longer variant (variant 5, also named RSRC2l) is a nonsense product with an additional exon 4 which includes a stop codon (Figure 3A). Overexpression of TRA2A in cells can regulate the alternative splicing of RSRC2 and result in the shift of RSRC2s to RSRC2l (Tieju Liu et al., 2017). We first examined whether the endogenous TRA2A influenced the alternative splicing of RSRC2. We transfected a siTRA2A to HEK293 cells to deplete the endogenous TRA2A and examined the shift of RSRC2. Upon the exhaustion of endogenous TRA2A, the abnormal splicing of RSRC2 (RSRC2l) decreased (Figure 3B). Then, the HEK293 cells were transfected with different amounts of TRA2A plasmid, respectively. The total RNA was extracted as the template for reverse transcription PCR (RT-PCR) to generate a cDNA library. We designed a pair of primers for RSRC2 amplification, and a pair of primers for GAPDH amplification was used as a control. The cDNA library was used as the template to amplify both the RSRC2 and GAPDH genes. There are two PCR products amplified: RSRC2s (204 bp), the main product and RSRC2l (282 bp), an abnormal splicing product with an additional exon 4 gene sequence inserted. With the increase of TRA2A, the amount of RSRC2l increased gradually in a dose-dependent manner (Figure 3C). These results validated that the abnormal splicing of RSCRC2 happened when the expression of TRA2A increased in the HEK293 cells. To detect whether the degradation of TRA2A mediated by E4B will affect the alternative splicing of RSRC2, the HEK293 cells were cotransfected with E4B and the PCR products of RSRC2 were detected by the same method. Compared to the control cells, which were transfected with neither E4B nor TRA2A (Figure 3D, lane 1), the RSRC2l gene product increased in the cells transfected with TRA2A (Figure 3D, lane 2). When transfected with both E4B and TRA2A, the RSRC2l gene product was reduced (Figure 3D, lane 3) compared with lane 2. The least RSRC2l gene product was obtained in the cells transfected with E4B but without TRA2A (Figure 3D, lane 4). We calculated the ratio of RSRC2l/RSRC2s according to the yields of the PCR products and the results are shown in Figure 3D (right panel). The ratio of RSRC2l/RSRC2s in the control cells was about 50% but increased to 70% in the TRA2A overexpressed cells. Nevertheless, the ratio decreased under 40% in the TRA2A overexpressed cells when cotransfected with E4B, indicating that E4B degraded the TRA2A. Compared to the control cells, the ratio in the E4B overexpressed cells was further decreased to 40%, meaning that endogenous TRA2A was degraded by E4B. Taken together, these results suggested that E4B reduced the alternative splicing of RSRC2 and affected its transcription products by mediating TRA2A degradation.

### E4B Slightly Mediated Degradation of Endogenous TRA2A and PYCR2 in Hepatocellular Carcinoma Cells

**FIGURE 4:**
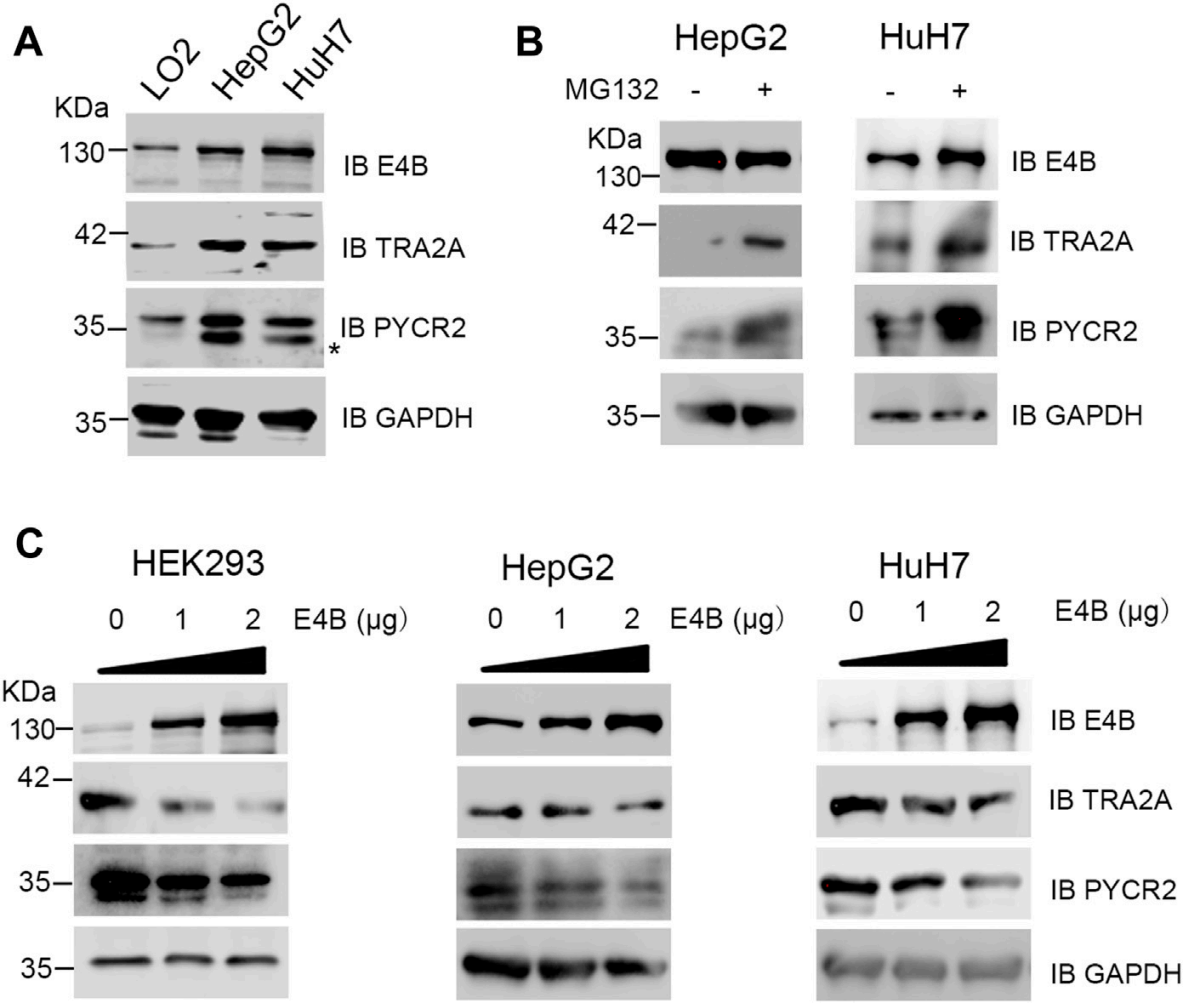
Degradation of endogenous TRA2A and PYCR2 in the HCC cells. **(A)** Expression of endogenous E4B, TRA2A, and PYCR2 in different cell lines. Asterisk might be a nonspecific binding with an anti-PYCR2 antibody. **(B)** Stability of TRA2A and PYCR2 disturbed by MG132 in the HCC cells. 10 μM MG132 was added to the HepG2 and HuH7 cells, and the endogenous expression of TRA2A and PYCR2 was detected with specific antibodies. **(C)** The increase of E4B degraded TRA2A and PYCR2 in the HEK293 cells but slightly in the HepG2 and HuH7 cells. Exogenous E4B was added to the HEK293 and the HCC cells in different amounts, and the degradation of endogenous TRA2A and PYCR2 was detected by specific antibodies.

TRA2A and PYCR2 are highly expressed in several cancer tissues and regarded as oncogenes or biomarkers (Chao Liu et al., 2017; Tang et al., 2018). However, E4B is also highly expressed in severe cancer cells. Therefore, we asked an interesting question: why can they overexpress in the same cells simultaneously since E4B induced the degradation of TRA2A and PYCR2? To answer this question, we detected the endogenous protein level of E4B, TRA2A, and PYCR2 in the HCC cell lines HepG2, HuH7, and normal human hepatocyte cell line LO2. Compared to the expression in LO2, the expression of endogenous E4B, TRA2A, and PYCR2 were significantly increased in the HepG2 and HuH7 cells (Figure 4A). These results are consistent with what has been reported. We further investigated the stability of TRA2A and PYCR2 in the HCC cell lines. We compared the protein levels in cells with or without MG132 treatment and found that the expression of both TRA2A and PYCR2 increased significantly when the cells were treated with MG132 (Figure 4B), demonstrating that the stability of TRA2A and PYCR2 depended on proteasome-related degradation. Considering other E3 ligases in the HepG2 and HuH7 cells, we could not judge whether the degradation of TRA2A and PYCR2 was related to E4B. To further study the role of E4B in the degradation of endogenous TRA2A and PYCR2, the HepG2, HuH7, and HEK293 cells were transfected with different amounts of exogenous E4B and the protein levels of TRA2A and PYCR2 were detected with specific antibodies. Consistent with the results in Figure 2A, the endogenous TRA2A and PYCR2 were significantly decreased with the increasing amount of E4B. To our surprise, even transfected with 2 μg E4B, both TRA2A and PYCR2 were slightly decreased in the HepG2 and HuH7 cells (Figure 4C). These results conflicted with the results in HEK293 cells, indicating that E4B mediated the degradation of endogenous TRA2A and PYCR2 slightly in the HCC cells. Further study is underway to reveal the degradation mechanism of TRA2A and PYCR2 in the HCC cells.

### E4B Interacted With Substrates *via* its Variable Region

**FIGURE 5:**
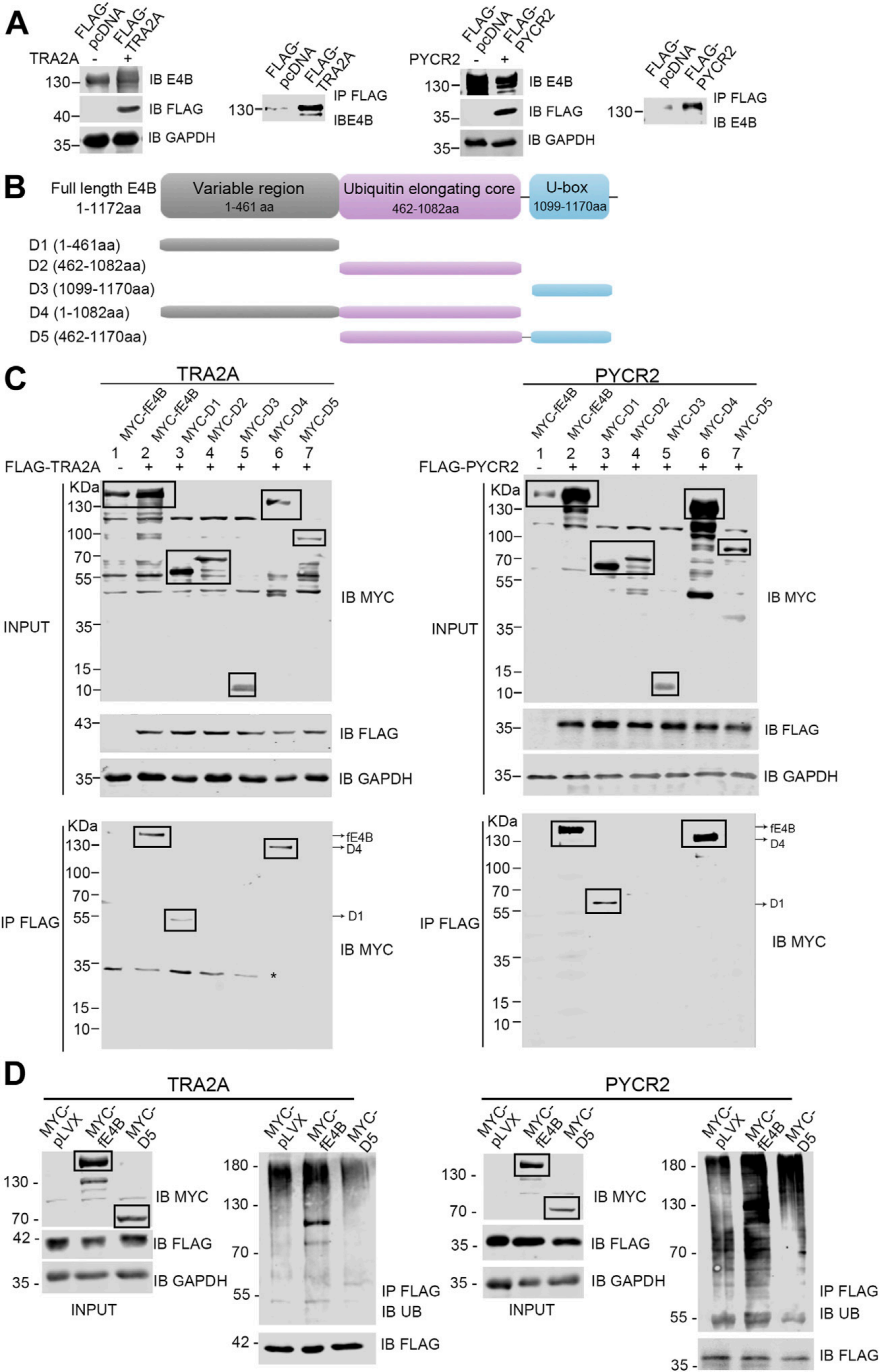
E4B interacted with substrates *via* its variable region. **(A)** Interaction between the full-length E4B and TRA2A and PYCR2. HEK293 cells were transfected with the full-length E4B (fE4B) with or without the transfection of exogenous FLAG-TRA2A and FLAG-PYCR2. Co-immunoprecipitation was carried out by an anti-FLAG antibody and the interaction was detected by an anti-E4B antibody. **(B)** Construction of E4B variants. Full-length E4B consists of a variable region (1-461 aa), a ubiquitin elongating core, and a U-box domain. Based on its sequence and structure, five truncated variants including D1 (1-461aa), D2 (462-1082aa), D3 (1099-1170aa), D4 (1-1082aa), and D5 (462-1170aa) were constructed. **(C)** Interaction between E4B truncated variants and FLAG-TRA2A and FLAG-PYCR2. The HEK293 cells were transfected with MYC-tagged full-length E4B and five variants are treated with MG132 (10 μM) for 4 h before harvesting. TRA2A and PYCR2 were pulled down with an anti-FLAG antibody and the interaction with E4B was detected with an anti-MYC antibody. Due to the nonspecific binding of the anti-MYC antibody, the targeted bands were boxed. Asterisk might be a nonspecific binding with anti-MYC antibody upon a nondenaturing condition. **(D)** Ubiquitination of TRA2A and PYCR2 was promoted by fE4B but not D5. The HEK293 cells were transfected with MYC-pLVX vector (control), MYC-fE4B, and MYC-D5, respectively. The ubiquitination of TRA2A and PYCR2 was detected by an anti-Ub antibody after pull-down by an anti-FLAG antibody upon a denaturing condition. The cells were treated with MG132 (10 μM) for 4 h before harvesting.

E4B contains a U-box domain, which is regarded as the domain for the binding with Ub-E2 conjugate (Hatakeyama et al., 2001). However, few reports showed how E4B interacts with its substrates. Ufd2p, the homolog of E4B in *S. cerevisiae,* catalyzed K29-linked polyubiquitin chain elongation *via* its two N-terminal loops located in the variable region (Chao Liu et al., 2017). In our study, we wanted to know which domain of E4B interacts with TRA2A and PYCR2. The HEK293 cells were cotransfected with the exogenous full-length E4B and FLAG-tagged substrates (TRA2A or PYCR2), and a co-immunoprecipitation assay was performed to identify the interaction between the full-length E4B with TRA2A or PYCR2. The HEK293 cell transfected with the full-length E4B and FLAG-tagged pcDNA empty vector (without the gene of TRA2A or PYCR2 inserted) was used as a control. The cell lysates were pulled down with anti-FLAG beads, and the interaction of substrates with E4B was detected by an anti-E4B antibody. The results showed that the full-length E4B could interact with either TRA2A or PYCR2 (Figure 5A), consistent with the result that E4B worked as an E3 in the ubiquitination of TRA2A and PYCR2.

E4B consists of three domains: a variable region (1-461 aa) at the N-terminus, a ubiquitin elongating core (462-1,082 aa), and a U-box domain (1,099-1,170 aa) at the C-terminus (Figure 5B). To further study the structure–function relationship between E4B and substrate, we constructed five MYC-tagged E4B truncated variants containing different domains based on the E4B sequence. We named these variants D1 (1-461 aa), D2 (462-1,082 aa), D3 (1,099-1,170 aa), D4 (1-1,082 aa), and D5 (462-1,170 aa), as shown in Figure 5B and detected the interaction with FLAG-tagged TRA2A or PYCR2 in the HEK293 cells. The full-length E4B (fE4B), D1, and D4 showed interaction with either TRA2A or PYCR2 (Figure 5C). D5, the variant that lacks variable region but contains ubiquitin elongating core and U-box domain, could not be recognized by TRA2A and PYCR2 (Figure 5C). These results indicated that the variable region is an indispensable domain for recognition of E4B with its substrate.

Although D5 did not show the interaction with TRA2A or PYCR2, it contains the U-box domain and can bind to Ub-E2 conjugate. We wanted to examine whether D5 would enhance the ubiquitination of TRA2A and PYCR2. The HEK293 cells were transfected with MYC-tagged pLVX plasmid, MYC-tagged fE4B, and MYC-tagged D5, respectively. Meanwhile, FLAG-tagged TRA2A or PYCR2 were cotransfected to all the groups. Ubiquitination was detected by immunoprecipitation of TRA2A or PYCR2 with anti-FLAG beads and immunoblotting with an anti-Ub antibody. Compared to the control cells (pLVX), the cells transfected with fE4B showed a significant increase of ubiquitination on TRA2A and PYCR2. However, the cells transfected with D5 did not increase the ubiquitination of TRA2A and PYCR2 (Figure 5D). Taken together, the variable region of E4B plays a key role in the interaction and recognition of substrates. Deleting the variable region of E4B will abolish the ubiquitination of its substrates.

## Discussion

E4B is known as a ubiquitin E4 enzyme and most research focused on its polyubiquitin elongation function on its substrates such as p53, ataxin-3, and Yap8 (Matsumoto et al., 2004; Wu et al., 2011; Ferreira et al., 2015). In our previous work, we engineered an orthogonal ubiquitin pathway (OUT) and identified over 100 potential substrates of E4B in the HEK293 cells (Zhao et al., 2012; Bhuripanyo et al., 2018). We found that TRA2A and PYCR2 are in the list but did not validate whether TRA2A and PYCR2 are bonafide substrates of E4B. TRA2A is highly expressed in breast cancer, glioma, and liver cancer, while PYCR2 is regarded as a prognostic biomarker in the HBV-related HCC (Tieju Liu et al., 2017; Tan et al., 2018; Gao et al., 2019). However, ubiquitination of TRA2A and PYCR2 has not been reported. In this study, we reported that E4B could mediate the ubiquitination of TRA2A and PYCR2 *in vitro* and HEK293 cells as an E3 ligase. Further study demonstrated that E4B degraded TRA2A and PYCR2 by forming K11- and K48- linked polyubiquitin chains on substrates. The overexpressed E4B affected alternative splicing of RSRC2 by mediating the degradation of TRA2A in the HEK293 cells (Figure 3D).

Since both E4B and its substrates, TRA2A and PYCR2, are highly expressed in many cancer tissues and cells, we wanted to know whether E4B can degrade TRA2A and PYCR2 efficiently in the same type of cancer cells. Intriguingly, although E4B was overexpressed in the HepG2 and HuH7 cells, it hardly degraded endogenous TRA2A and PYCR2, even transfected with exogenous E4B (Figure 4C). These results conflicted with those in the HEK293 cells, suggesting that the mechanism of degradation of TRA2A and PYCR2 mediated by E4B is different in cancer cells. For example, deubiquitinating enzymes (DUBs) play a role in cleaving the Ub chain and decreasing the ubiquitination of substrates in the cancer cells. It has been reported that USP2a, USP7, USP21, and USP22 are highly expressed in the HCC tissue or cells and promote tumor development (Cai et al., 2015; Li et al., 2018; Ling et al., 2020; Xiong et al., 2021). However, further study is needed to reveal the mechanism in the HCC cells.

So far, it is unknown of the E4B structure and how E4B interacts with its substrates. Ufd2, the yeast homolog of E4B, consists of a highly variable N-terminal region, a core region domain, and a U-box domain (Tu et al., 2007). Based on the structural similarity, we constructed five E4B truncated variants and investigated the interaction between different domains of E4B and substrates. In addition to wtE4B, only D1 and D4 variants showed interaction with TRA2A and PYCR2, indicating that E4B was bound to its substrates *via* its variable region. These results are consistent with those of a recent study on Ufd2. In their work, Ufd2 was bound to the substrate GFP-Ub *via* its two N-terminal loops located in the highly variable region and played as an E4 enzyme *in vitro* (Chao Liu et al., 2017).

In conclusion, our study reported the ubiquitination and degradation of TRA2A and PYCR2 in different cell lines. The variable region of E4B was indispensable to interact with its substrates. The degradation of TRA2A and PYCR2 in the HEK293 cells was quite different from that in the HCC cells. These results revealed other mechanisms associated with the ubiquitination of TRA2A and PYCR2 in the HCC cells. The mechanism of why E4B does not degrade TRA2A and PYCR2 effectively needs further study in the future.

## Data Availability

The original contributions presented in the study are included in the article/Supplementary Material. Further inquiries can be directed to the corresponding author.

## Supplementary Material

Supplementary Figure S1 Ubiquitin transfer assay of E1, E2, E3, and substrates. E1, E2, E3, and substrates were sequentially added into the mixed reaction to show each component by detecting the HA-Ub complex with an anti-HA antibody upon a nondenaturing condition. The ubiquitination of TRA2A and PYCR2 was also detected with an anti-FLAG antibody upon a denaturing condition.
