# Supplementary figures and images for: Differential Degradation of TRA2A and PYCR2 Mediated by Ubiquitin E3 Ligase E4B
Source: Front Cell Dev Biol. 2022 May 20;10:833396. doi: 10.3389/fcell.2022.833396 (PMC9163560; doi:10.3389/fcell.2022.833396)

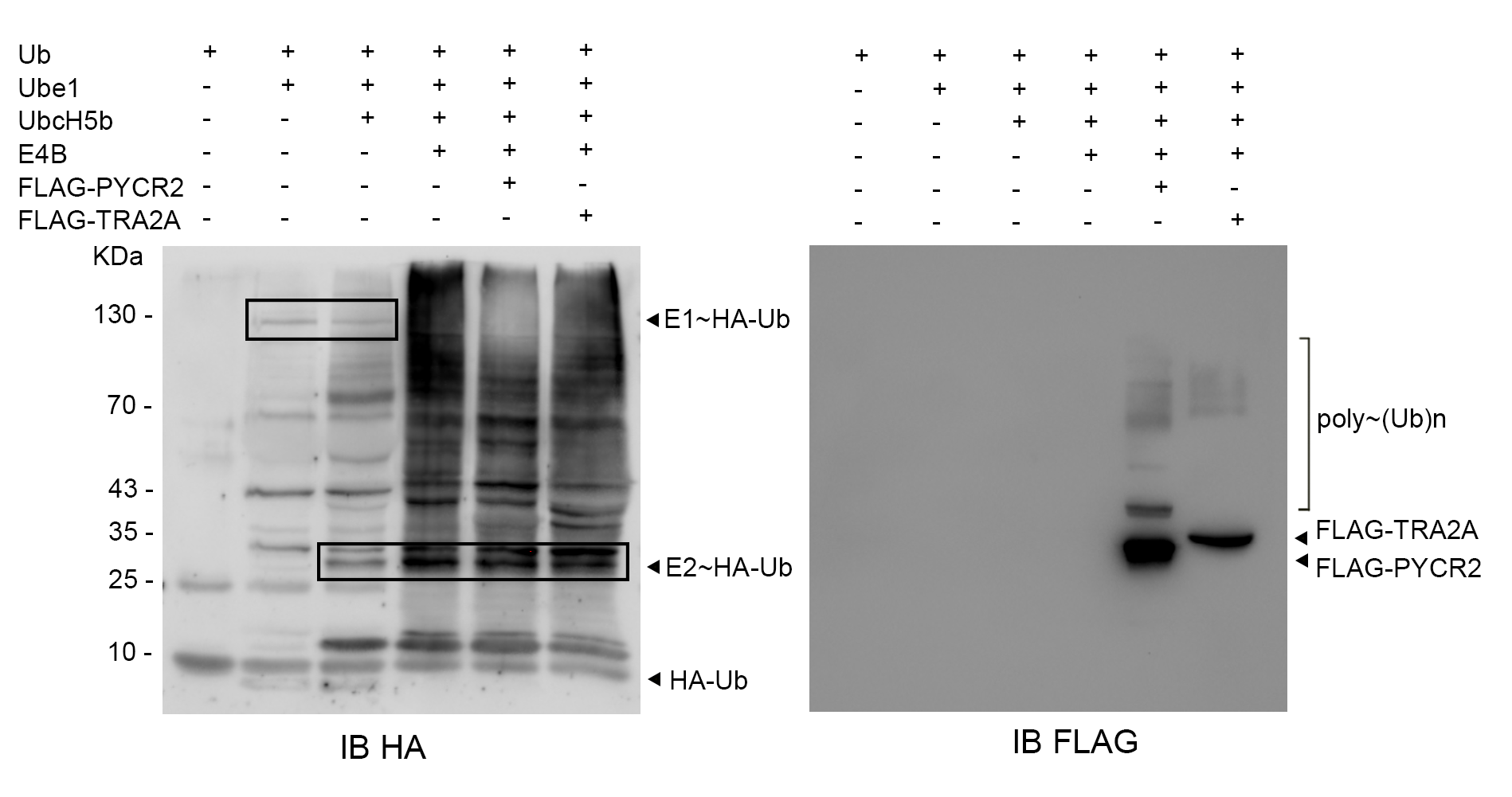

Supplement: Supplementary file 1 [file Image1.TIF]
